# Anti-Biofouling and Desalination Properties of Thin Film Composite Reverse Osmosis Membranes Modified with Copper and Iron Nanoparticles

**DOI:** 10.3390/ma12132081

**Published:** 2019-06-28

**Authors:** M. Armendariz Ontiveros, Y. Quintero, A. Llanquilef, M. Morel, L. Argentel Martínez, A. García García, A. Garcia

**Affiliations:** 1 Instituto Tecnológico de Sonora. 5 de Febrero 818 Sur, Sonora 85000, Mexico; 2 Advanced Mining Technology Center (AMTC), Universidad de Chile, Santiago 8370451, Chile; 3 Facultad de Ciencias Naturales, Departamento de Química y Biología, Universidad de Atacama, Copiapó 1531772, Chile; 4 Instituto Tecnológico del Valle del Yaqui, C. 600, Block 611, Sonora 85275, Mexico; 5 Laboratorio de Síntesis y Modificación de Nanoestructuras y Materiales Bidimensionales, Centro de Investigación en Materiales Avanzados S.C. Parque PIIT, Apodaca Nuevo León 66628, Mexico

**Keywords:** biofouling, RO membranes, Cu nanoparticles, Fe nanoparticles, desalination

## Abstract

The anti-biofouling and desalination properties of thin film composite reverse osmosis membranes (TFC-RO), modified by the incorporation of copper and iron nanoparticles, were compared. Nanoparticles of metallic copper (CuNPs) and an iron crystalline phase mix (Fe and Fe_2_O_3_, FeNPs) were obtained by oxide-reduction-precipitation and reduction reactions, respectively, and characterized by X-ray diffraction (XRD) and transmission electron microscopy (TEM) techniques. Modified membranes (PA+0.25Cu-PSL and PA+0.25Fe-PSL) were obtained by incorporating these nanoparticles during the interfacial polymerization process (PI). These membranes were characterized by scanning electron microscopy (SEM), energy-dispersive X-ray spectroscopy (EDX), atomic force microscopy (AFM), and contact angle measurements. Bactericidal tests by a Colony Forming Unit (CFU) were performed using *Escherichia coli*, and anti-adhesion properties were confirmed by fluorescence microscopy estimating the percentage of live/dead cells. The permeate flow and rejection of salts was evaluated using a crossflow cell. An increase of the membrane’s roughness on the modified membrane was observed, influencing the desalination performance more strongly in the presence of the FeNPs with respect to the CuNPs. Moreover, a significant bactericidal and anti-adhesion effect was obtained in presence of both modifications with respect to the pristine membrane. An important decrease in CFU in the presence of modified membranes of around 98% in both modifications was observed. However, the anti-adhesion percentage and reduction of live/dead cells were higher in the presence of the copper-modified membrane in comparison to the iron-modified membrane. These facts were attributed to the differences in antimicrobial action mechanism of these types of nanoparticles. In conclusion, TFC-RO membranes modified by the incorporation of CuNPs during PI represent one alternative material to attend to the biofouling impact in the desalination process.

## 1. Introduction

Reverse osmosis (RO) desalination is a possible alternative that reduces water scarcity rates worldwide [1]. RO had an annual contracted capacity of 2.2 million m^3^ d^−1^ in 2017 [2]. The main RO membranes on the market are based on a polyamide (PA) thin film composite (TFC) synthesized by an interfacial polymerization process, which consists of a combination of an aqueous m-phenylenediamine (MPD) solution and an organic trimesoyl chloride (TMC) solution [3]. However, TFC-RO membranes face a biofouling problem, which involves the adhesion, growth, reproduction, and proliferation of microorganisms onto the membrane surface [4], causing permeate flux reduction, increasing the operating pressure, and therefore increasing process costs [5]. 

In order to avoid biofouling in the membranes, researchers have incorporated, on the PA layer, different inorganic nanoparticles (NPs) with antimicrobial properties, such as Ag, TiO_2_, and ZnO, among others [6,7,8]. However, some of these NPs are quite expensive because of the extraction process from the earth and are difficult to synthesize. For this reason, it is necessary to use antimicrobial NPs that are easy to obtain from nature, easy to synthesize, and are easily added to TFC membranes without significant costs to the desalination process, such as copper and iron nanoparticles [9,10,11]. 

These two types of NPs have a high biocide effect against a large diversity of microorganisms by different mechanisms. Since iron nanoparticles reduce nutrient transport into bacteria cells, they cause nutritional imbalances and metabolic weakness in bacteria. In addition, they produce oxidative stress by generation of some reactive oxygen species (ROS) with free radicals (Fenton or Fenton-like reaction), damaging cellular proteins, lipids, and DNA, and eventually leading to bacteria death [12,13]. On the other hand, the copper nanoparticles act by releasing ions, thereby destroying vital parts of the bacteria cells. In addition, they also generate ROSs that damage the bacteria’s DNA, killing them [14]. 

Despite this, it is important to note that the bactericidal effect of copper and iron can be influenced by the type nanoparticles, either metallic or oxide. In the case of iron nanoparticles, although both types possess antimicrobial properties, metallic nanoparticles are more bactericidal than iron oxides. In the presence of dissolved oxygen, metallic iron nanoparticles can generate a Fenton reaction, producing hydrogen peroxide in addition to ROS [15]. 

Likewise, differences in the biocidal effect between metallic and oxide copper nanoparticles can also be observed. The biocidal mechanism of copper oxide nanoparticles has been associated with ion release, since these are highly soluble [16]. Moreover, the redox cycle, caused between the ionic species Cu (I) and Cu (II), generates reactive superoxide species that contribute to the degradation of biomolecules [17]. However, metallic copper nanoparticles could have a greater biocidal effect considering that, in addition to the release of copper ions, the mechanism for the biocidal effect also corresponds to the generation of greater reactive oxygen species (ROSs). The absorption of this nanoparticle by microorganisms leads to a series of interactions at the level of structural proteins, organelles, and DNA, contributing to oxidative stress within the cell [18,19].

Thus, the modification of RO membranes with metallic or oxide nanoparticles in order to improve the anti-biofouling effect is not a trivial issue. Metallic nanoparticles could favor the effect with respect to the oxidized species.

In this regard, some proposals about TFC membranes modified with copper and iron nanoparticles have been reported in recent years [20,21,22,23,24]. For this, development of several strategies to modify TFC RO membranes with these nanoparticles have been proposed; these strategies include the dip-coating method, which is one alternative that was widely reported [12,21,22,23]. 

For example, Ben-Sasson et al. [21] functionalized a TFC RO membrane with elemental Cu nanoparticles (CuNPs) by using a dipping method and obtained a 80%–95% reduction in the bacteria adhesion onto the membrane surface. Moreover, Ben-Sasson et al. [22] also coated a commercial TFC RO membrane with in situ formed CuNPs and obtained a flux increment (20%) and a reduction in bacterial attachment onto the membrane surface (90%). Nevertheless, these modification methods have shown low stability as their principal disadvantage.

In this regard, modifications during the interfacial polymerization process represent another alternative. For example, Garcia et al. [20] incorporated CuONPs onto the PA layer of a TFC membrane during the interfacial polymerization process and obtained a high anti-adhesion and bactericidal effect, without affecting the membrane performance. In addition, Rodriguez et al [24], added in situ, during the interfacial polymerization process, a Cu-(m-phenylenediamine) oligomer complex within the PA layer of the TFC-RO membrane. They found a 99% bacterial reduction onto membranes and obtained an increment of 25% in permeate flux. 

Thus, although the incorporation of different types of copper nanoparticles (CuO and oligomers) during the interfacial polymerization process into the PA layer have been explored, the use of metallic copper nanoparticles (CuNPs) during this process has not been studied yet. Therefore, significant differences in anti-biofouling effects could be observed in the presence of Cu nanoparticles with respect to others (such as CuO nanoparticles), considering the differences in the bactericidal mechanism of these types of nanoparticles. Thus, the incorporation of metallic copper nanoparticles (CuNPs) could have favorable performance with respect to the options explored at the moment. 

On the other hand, TFC membranes modified with iron nanoparticles have been infrequently studied. For example, Armendariz et al. [12] modified a commercial membrane surface with FeNPs by using a dip coating method, obtaining a reduction of 90% in the formation of a biofilm cake layer, but affecting the desalination performance membrane. In this case, the modification method was dip coating, which has disadvantages due to the low stability described above.

Meanwhile, only one approach to modify with iron nanoparticles during the interfacial polymerization process was done by Homayoonfal et al. [25], who incorporated iron oxide nanoparticles (Fe_3_O_4_ NPs) into the PA layer membrane, observing an enhance in the membrane flux. Nevertheless, in this case, the anti-biofouling effect was not studied.

In this way, the incorporation of nanoparticles in the PA layer membranes during the interfacial polymerization process is an alternative method of modifying RO membranes. However, the incorporation of metallic copper (CuNPs) and iron (FeNPs) nanoparticles into the PA layer during this interfacial polymerization process has not been studied yet. 

Thus, considering that both nanoparticles have significant antimicrobial properties, but differences in the antimicrobial action mechanism have been reported between them, their incorporation into TFC-RO membranes could be an interesting comparative study on their influence on anti-biofouling effects and the desalination performance of these modified membranes. Therefore, this research aims are to modify RO membranes by incorporating metallic Cu and Fe nanoparticles into the PA layer during the interfacial polymerization process in order to enhance anti-biofouling and anti-adhesion properties and study their behavior in the desalination performance.

## 2. Materials and Methods 

The following chemicals were used: ferric nitrate (Fe(NO_3_)_3_·9H_2_O; > 99%, ACS, Fermont, Monterrey, Mexico), ethanol (C_2_H_5_OH; > 99%, J.T. Baker, Mexico), sodium borohydride (NaBH_4_; > 99%, Fluka, Mexico city, Mexico) used for FeNP synthesis. L-ascorbic acid (C_6_H_8_O_6_; > 99%, Sigma Aldrich, Darmstadt, Germany), copper sulphate (CuSO_4_·5H_2_O; > 99%, ACS, Fermont, Monterrey, Mexico), etilenglicol (C_2_H_6_O_2_; > 99.5%, Merck, Nottingham, UK), sodium hydroxide (NaOH; > 50% p/v, Winkler, Santiago, Chile) used for CuNP synthesis. Polysulfone Udel P-3500 MB7 (in pellet form, Solvay Advanced Polymers), 1-methyl-2 pyrrolidinone (NMP, > 99.5%, Sigma Aldrich, Darmstadt, Germany), and *N*,*N*dimethylformamide (DMF, > 99%, Sigma Aldrich, Darmstadt, Germany) were used to make the polysulfone (PSL) sheet used as membrane support. *m*-phenylenediamine (MPD) and trimesoyl chloride (TMC) (Sigma Aldrich, Darmstadt, Germany), NaOH (> 97%, Sigma Aldrich, Darmstadt, Germany), and *n*-hexane (> 95%, Sigma Aldrich, Darmstadt, Germany) were used for PA monomer synthesis.

### 2.1. Synthesis of CuNPs and FeNPs

CuNPs were obtained following Liu et al. [26] by an oxide-reduction-precipitation reaction. The type of stirring and wash processes were modified. Into a solution of CuSO_4_·5H_2_O in ethylene glycol (40 g L^−1^), 17 g of ascorbic acid and 250 mL of Sodium hydroxide were added to 0.01 M; this solution was maintained under sonication to 55 °C and at 53 KHz for 1 h. Once the reaction was complete, a red precipitate was obtained and separated from the supernatant. The reddish solid was washed with a 2% solution of MPD and dried in a vacuum oven (Thermo Fisher Scientific, Madrid, Spain) for 8 h at 60 °C.

FeNPs were synthetized following Baltazar et al. [27] and Arancibia-Miranda et al. [28,29]. NaBH_4_ aqueous solution (4.48 g L^−1^) was added to a Fe(NO_3_)_3_·9H_2_O aqueous solution (4.00 g L^−1^) and kept under agitation for 1 h at 25 °C. After this time, the nanoparticles were washed with distilled water and acetone. 

### 2.2. Characterization of CuNPs and FeNPs

X-ray Diffraction (XRD) analysis was performed in order to study the crystallinity of the CuNPs and FeNPs. The XRD tests were done in a diffractometer (Bruker D8 Advance) (Bruker, Madison, USA) with CuKα radiation. Transmission electron microscopy (TEM) was used to observe the morphology and size of the CuNPs and FeNPs. TEM micrographs were taken in a microscope (Tecnai F20 FEG-S/TEM) (FEI Tecnai, Texas, USA) operated at 200 kV. The particle size distribution was obtained by processing TEM images with the DigitalMicrograph software (Gatan, Pleasanton, USA) for a population of at least 100 particles. 

### 2.3. Synthesis of TFC RO Membrane with CuNPs and FeNPs

TFC membranes modified by incorporating CuNPs and FeNPs were prepared following the method of Garcia et al. [6,20]. A polysulfone (PSL) solution was prepared by dissolving polysulfone pellets (16 wt%) in a 4:1 DMF/NMP mixture, stirred for 2 h at 50 °C. The PSL membrane support was prepared by spreading the polymer solution uniformly on a glass plate using a film-casting knife (BYK, Geretsried, Germany) with a clearance set at 200 μm. Then, the membrane support was submerged in a coagulation bath after 1 min and washed exhaustively with distilled water in order to get rid of any residual solvent under laboratory conditions. 

PA and modified PA with CuNPs and FeNPs were prepared using the interfacial polymerization reaction of the aqueous phase of the MPD and the organic phase of the TMC on the porous PSL substrate. The PSL support was placed for 2 min in a 2 wt% aqueous MPD solution containing 0.05 wt% NaOH. Next, the support was immersed in a 0.2 wt% TMC solution in hexane for 1 min to allow interfacial polymerization. Then, the membrane was cured in an air circulation oven (Thermo Fisher Scientific, Madrid, Spain) at 75 °C for 8 min. After, it was washed with distilled water and dried at room temperature for 24 h. The modified membrane was prepared by adding CuNPs (0.25 wt%) and FeNPs (0.25 wt%) homogeneously dispersed in an MPD solution with an ultrasonic bath to prepare the CuNPs and FeNP-entrapping PA layers on the porous PSL supports, referred to as PA+0.25Cu-PSL and PA+0.25Fe-PSL, respectively.

### 2.4. Characterization of Membranes Modified by Incorporation of CuNPs and FeNPs

The surface morphology of the membranes and their elemental analysis were examined by SEM-EDX-CL (FEI Quanta 250) (Thermo Fisher Scientific, Madrid, Spain). The SEM measurements were performed at low vacuum conditions with a Helix detector (Thermo Fisher Scientific, Madrid, Spain) at 5 kV. The samples were deposited on a lacey form over carbon, with 300 mesh Cu grids, prior to examination. Changes in the surface roughness of the membranes were examined by AFM (NanoWizard 3 BioScience, JPK Instruments) (Bruker, Madison, USA). Hydrophilicity analysis was realized by the contact angle measured by placing a drop of ultra-pure water on the surface membrane and performing a drop-shape analysis. Contact angle images were captured using a Digi-Microscope camera (JENOPTIK, Fremont, USA), a zoom lens of 500X, and a resolution of 11 µm/pixel.

### 2.5. Anti-Biofouling Test

Escherichia coli (*E. coli*) was used to evaluate the bactericidal effect of membranes, following the methodologies of Garcia et al. [6,20] and Rodriguez et al. [24]. Bacteria were inoculated in a solution (tryptone soya broth, TSB) and incubated in a shaking incubator at 30 °C. Then, the bacterial solution was centrifuged to remove nutrients at 3000 rpm for 10 min and washed in phosphate-buffered saline (PBS). The prepared bacteria were diluted in PBS to obtain a bacteria concentration of about 1 × 10^7^ cell/mL. The membrane samples were cut (2 × 2 cm^2^) and sterilized using ultraviolet radiation for 30 min. Then, membranes were immersed into 10 mL of the bacterial solution and incubated in the shaking incubator at 30 °C for 4 h. After this time, and to learn the bactericidal effect of the modified membranes in the medium, the bacterial solutions were serially diluted with PBS until achieving a concentration of 1 × 10^−3^ cell/mL. Later, 1 mL of the dilutions was spread, by triplicate, onto the surface of petri-dishes containing LB agar, and incubated for 24 hours, at 35 °C. Then, the colonies were counted to estimate the number of viable *E. coli* remaining in the suspensions. 

The bacterial adhesion onto membranes was assessed by the live and dead method. After being incubated for 4 h, the membranes were removed from the suspension and gently rinsed with a 0.85 wt% NaCl solution in water to remove the unadhered bacteria. Membranes were cut (1 cm^2^) in order to evaluate bacterial viability using a bacterial viability kit (Molecular Probes, LIVE/DEAD BacLight L7007) (Thermo Fisher Scientific, Madrid, Spain). Results were analyzed by epifluorescence microscope (Zeiss, AxioLab A1, Zeiss, Darmstadt, Germany) with a 100X objective. Live and dead cells were counted, and the results were statistically processed in order to estimate the bactericidal and anti-adhesion effects on the membrane surface. 

### 2.6. Statistical Analysis

For the colony forming units (CFU), live and dead cells, the theoretical t-student probability distribution of p ≤ 0.001 was used, using a sample size of n < 30. For all analyses, the professional statistical software STATISTIC 8.5 (OriginLab, Northampton, USA) was used.

### 2.7. Desalination Performance Test

Permeate flux and salt rejection of the membranes were evaluated using a cross flow test cell, a feed solution of 1000 mg/L NaCl at 300 psi, and an effective membrane area of 33 cm^2^, following the previous report [20,24]. The experiments were carried out at a temperature of 25 ± 1 °C. During 60 min, the permeate flux test of the membranes was calculated and the solute rejection was measured from the feed and the permeate solution concentrations. The conductivity of these two solutions was measured to obtain their respective concentration values.

## 3. Results and Discussions

### 3.1. Characterization of Cu and Fe Nanoparticles

**Figure 1:**
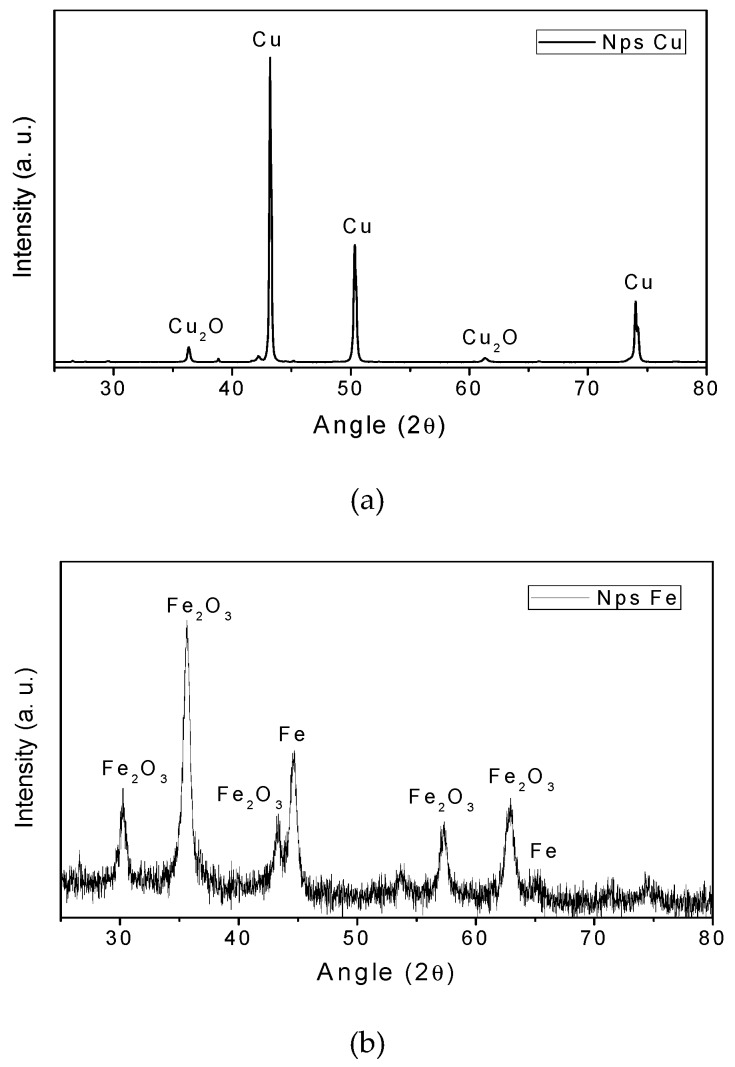
XRD patterns of the samples. (**a**) CuNPs; (**b**) FeNPs.

The XRD results of the CuNPs and FeNPs synthesized are shown in Figure 1. Figure 1a shows a copper compound, where the crystalline system of CuNPs is cubic. Characteristic picks appear at 2θ = 43.3°, 50.4° and 74.1°, corresponding to metallic Cu [20,30,31]. In addition, the spectrum shows peaks at 2θ = 36.44° and 61.41°, corresponding to Cu_2_O [22,32] in low concentrations. This result may be attributed to the easy oxidation of CuNPs in the atmosphere [23,24,33,34]. 

On the other hand, in Figure 1b it is possible to observe the presence of iron mix compounds, where the distinctive points of Fe appear at 2θ = 44.5° and 65.0°, confirming the crystalline structure of the FeNPs [14,30]. In addition, another phase corresponding to iron oxide (III) (Fe_2_O_3_) was found with peaks at 2θ = 30.3°; 35.6°; 43.5°; 57.2°, and 62.7° [29]. The Fe_2_O_3_ presences is probably due to the easy oxidation of FeNPs in air [32].

**Figure 2:**
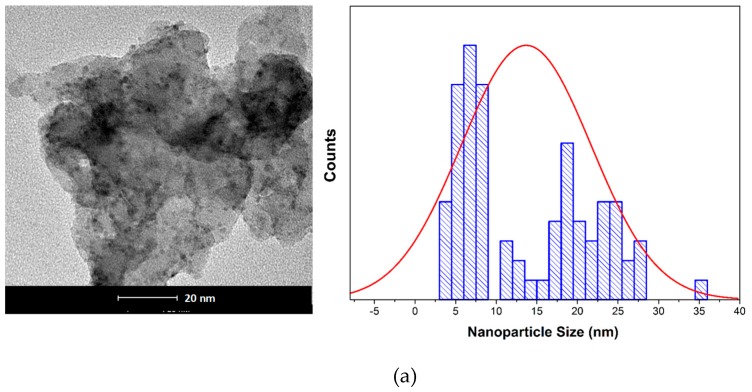

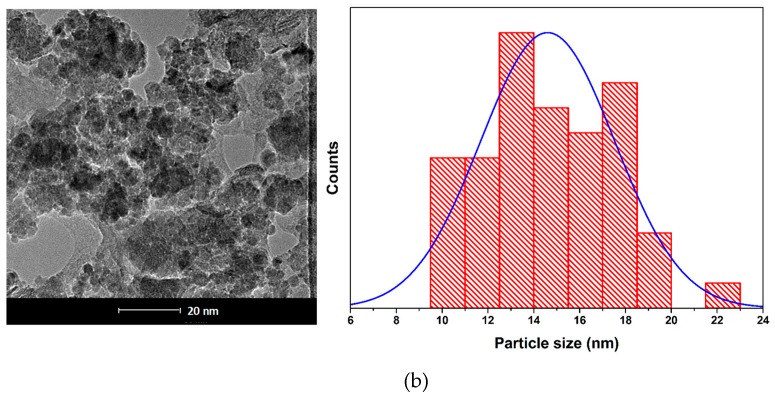
Transmission electron microscopy images and particle size distributions. (**a**) CuNPs; (**b**) FeNPs.

In Figure 2, TEM images and particle size distributions of the nanoparticles are shown. The TEM image of Figure 2a exhibits the formation of nanoparticles of CuNPs embedded in the stabilized agent used in the washing process. The particle size distribution of CuNPs shows two populations between 3–8 nm and 13–27 nm, with the mean particle size around 13.5 ± 8.0 nm. In Figure 2b, it is possible to observe spherical shapes for FeNPs with mean particle size distributions around 14.6 ± 1.2 nm. An amorphous matrix is observed around the nanoparticles related to the residual reductant agent used in the synthesis. It is showed that both CuNPs and FeNPs have similar particle sizes. Also, both NPs have a very small particle size (< 30 nm), which could improve the NP dispersion into the PA layer by increasing the coverage area of the NPs into the membrane, causing higher contact with bacteria, which could increase the biocide effect of the membranes [35]. 

### 3.2. Characterization of Modified and Unmodified TFC RO Membranes

**Figure 3:**
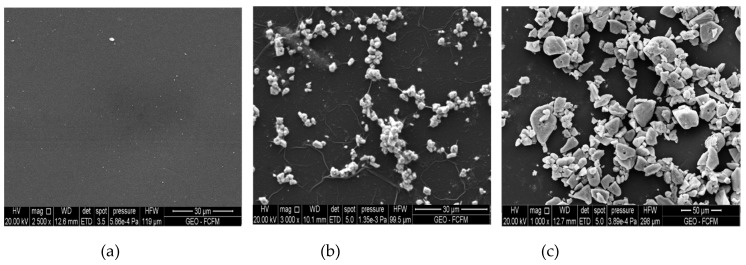
SEM images of the membrane surface (**a**) PA-PSL; (**b**) PA+0.25Cu-PSL; (**c**) PA+0.25Fe-PSL.

Figure 3 shows the SEM images of the PA-PSL, PA+0.25Cu-PSL, and PA+0.25Fe-PSL membrane surfaces (Figure 3a, b and c, respectively). It exhibits the morphological surface of pristine membrane without nanoparticles (Figure 3a). Moreover, certain agglomeration of the nanoparticles on modified membranes can be observed (Figure 3b and c). 

**Figure 4:**
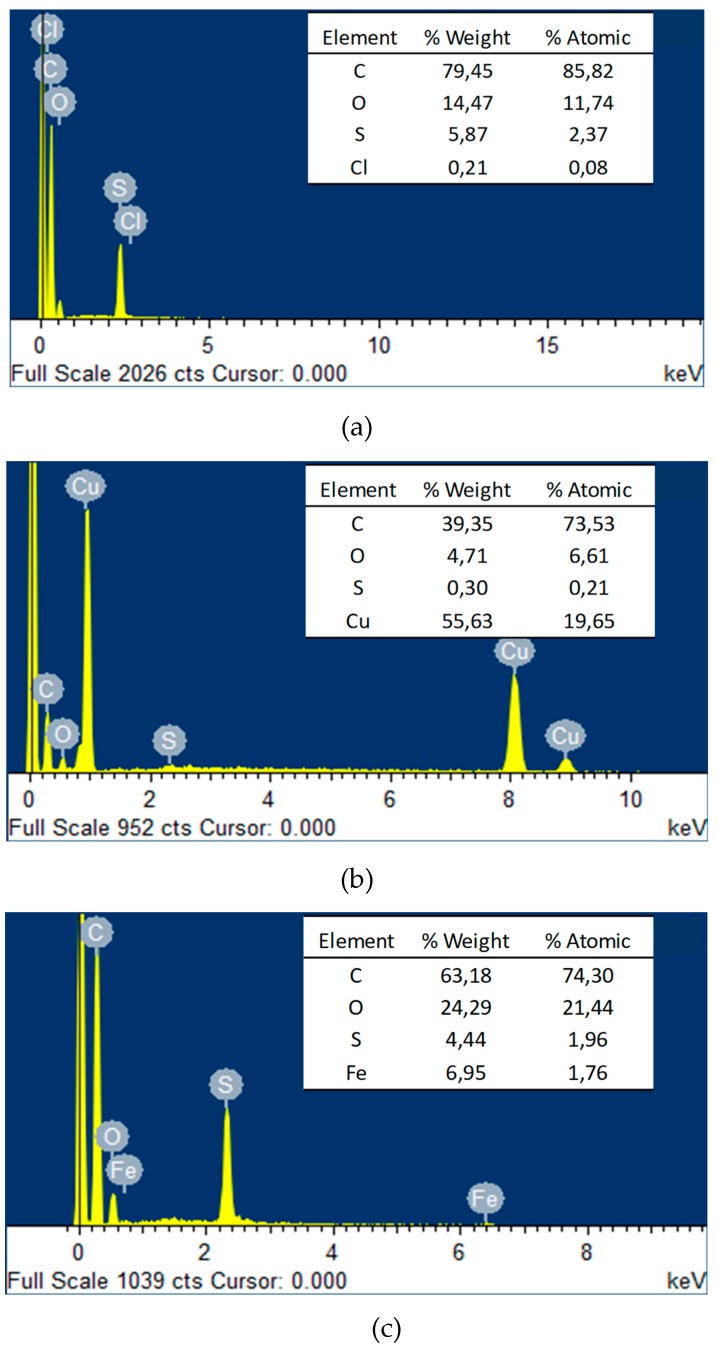
EDX analysis of membranes surface (**a**) PA-PSL; (**b**) PA+0.25Cu-PSL; (**c**) PA+0.25Fe-PSL.

The EDX results of membranes surfaces are shown in Figure 4, confirming the presence of Cu (Figure 4b) and Fe (Figure 4c) in the active layer of the PA+0.25Cu-PSL and PA+0.25Fe-PSL membrane. In the table (inside of Figure 4a–c), it is possible to observe quantitative results of the analyses, evidencing that C, O, and Cu were the main elements on the PA+0.25Cu-PSL membrane surface (Figure 4b) and C, O, and Fe were on the PA+0.25Fe-PSL membrane surface (Figure 4c).

Table 1 shows the roughness and contact angle values. In this table it is possible to observe significant changes in the roughness of modified membrane surfaces with respect to the pristine membrane. These values could be the result of the agglomeration tendency of NPs on the surface membranes in accordance with those observed in the SEM image (Figure 3). The PA+0.25Cu-PSL and PA+0.25Fe-PSL membranes’ roughness showed highly significant differences, being rougher in the presence of FeNPs than CuNPs. It has been reported that iron nanoparticles have a high agglomeration tendency, which can be an impediment for their good uniform dispersion on the membrane surface [12,36]. This can be due to the high surface energy, magnetic force, and electrostatic interaction of these FeNPs, which could lead to cluster formation [12]. This result could increase bacterial adhesion onto the modified membranes, considering that microorganisms are prone to attach in rougher topologies, reducing the anti-biofouling effect [6,20,24,37]. 

**Figure 5:**
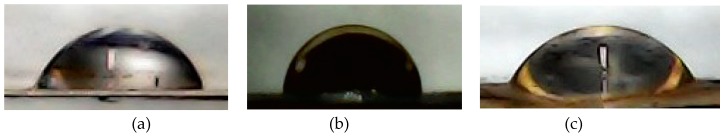
Images of water drops on membranes: (**a**) PA-PSL; (**b**) PA+0.25Cu-PSL; (**c**) PA+0.25Fe-PSL.

On the other hand, contact angle results are also presented in Table 1 and visualized in Figure 5. No significant influences on this parameter in the presence of the PA+0.25Fe-PSL membrane with respect to unmodified membrane (PA-PSL) were observed. In this regard, the highest contact angle was found for the PA+0.25Cu-PSL membrane (> 33% than PA-PSL). Thus, a significant decrease in the hydrophilicity character for the PA+0.25Cu-PSL membrane was observed [38], which is in concordance with previous reports for copper-modified membranes [20,24]. This fact suggests an alteration to the affinity of fouling materials for membrane surfaces [39] and a reduction of the solubility of water on the membrane surface. 

### 3.3. Anti-Biofouling Effect

**Figure 6:**
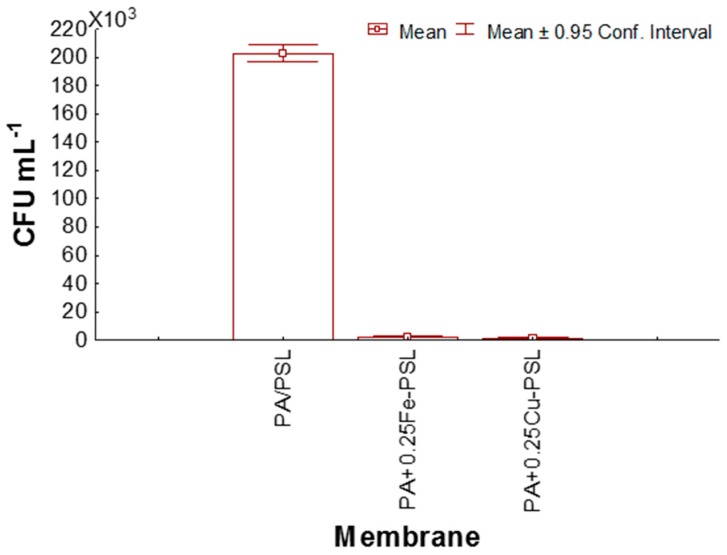
Bactericidal effect of the membranes on E. coli.

Figure 6 shows the bactericidal effect of membranes in the medium, in which a highly significant decrease in CFU is observed in the presence of modified membranes with respect to the unmodified membrane. However, there was no statistically significant difference between both modified membranes. The reduction of the modified membranes with respect to the unmodified membrane was > 99% for PA+0.25Cu-PSL and >98% PA+0.25Fe-PSL. This result evidences the great biocide effect of the CuNPs and FeNPs.

**Figure 7:**
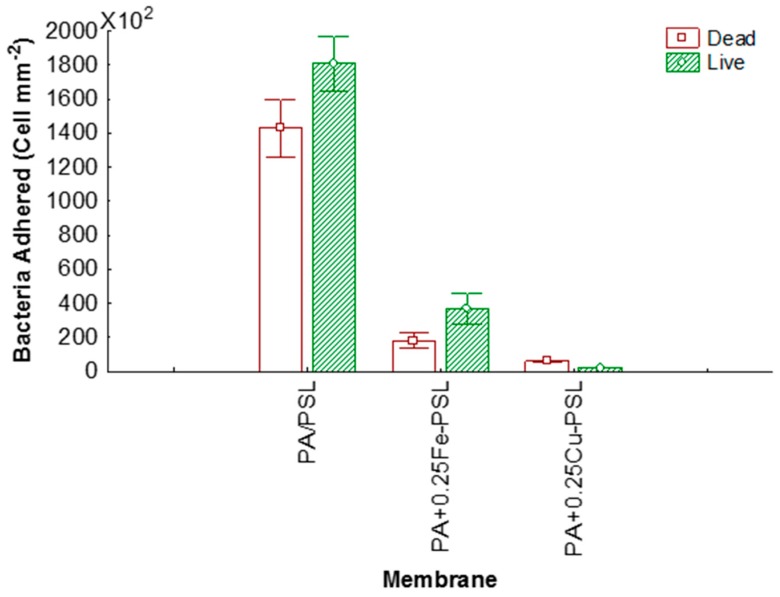
Cells of *E. coli* per area of the membranes.

On the other hand, the bacteria that adhered onto membranes are presented in Figure 7. It is possible to observe a highly significant reduction of the bacteria adhering onto the modified membranes with respect to the unmodified membrane. The total anti-adhesion percentage of PA+0.25Cu-PSL and PA+0.25Fe-PSL membranes were 97% and 83%, respectively, demonstrating a better effect with modification via CuNPs. Moreover, a higher reduction of live cells on the membrane’s surface was observed for PA+0.25Cu-PSL with respect to PA+0.25Fe-PSL.

These results may be attributed to the higher reactivity of CuNPs provided by the release of toxic Cu^+2^ ions, in addition to the generation of reactive oxygen species (ROS). CuNPs produce ROS in the medium that damages bacterial DNA causing bacterial death [14], and the release of ions increases intracellular ROS in the bacteria, thereby inflicting its death [40]. Meanwhile, the bactericidal power of FeNPs can be attributed only to ROS generation through the Fenton reaction, provoking bacterial death [12,13,32,41]. Previous reports have also described how CuNPs produce four ROS types [superoxide radical (O^2−^), the hydroxyl radical (⋅OH), hydrogen peroxide (H_2_O_2_), and singlet oxygen (O^2^)] [35] compared to only three ROS types (O^2−^, OH and H_2_O_2_) produced by FeNPs [42], which can is associated with the best performance of the modified membranes with copper versus iron. 

It must be also emphasized that the incorporated iron nanoparticle into the membrane was an iron crystalline phase mix (Fe and Fe_2_O_3_). Although both iron species possess antimicrobial properties, metallic nanoparticles make it more bactericidal than iron oxides. In the presence of dissolved oxygen, metallic nanoparticles can generate a Fenton reaction, producing hydrogen peroxide in addition to ROS [12]. the presence of Fe_2_O_3_ can likely limit the beneficial effect of this modification. 

Based on above description, the copper-modified membrane gives a better anti-biofouling effect than the iron and provides less adhesion, thereby proving less important to the roughness effect.

### 3.4. Desalination Performance

Table 2 shows the permeate flux and salt rejection percentage of the modified membranes and the pristine membrane. A slight decrease of permeate flux and rejection percentage on the modified membrane with CuNPs respective to the unmodifed membrane was observed. However, this effect is more detrimental in the presence of FeNPs. This fact can be due to the formation of defects in the polyamide layer by the incorporation of nanoparticles, in concordance with an increase of the roughness observed on a modified membrane promoted by the agglomeration of the nanoparticles on the membrane surface; this phenomenon was more strongly observed in the presence of FeNPs. Thus, as a consequence of this modification, the nanoparticles in the active membrane layer may be blocking the preferential flow channel water, reducing the permate capacity [43]. 

## 4. Conclusions

The adding of CuNPs and FeNPs within the PA layer of the TFC-RO membranes during the interfacial polymerization process increased the membrane roughness, thereby affecting the desalination performance, and this effect was more remarkable in the membrane modified with FeNPs. However, a significant bactericidal and anti-adhesion effect on the membrane was achieved. The beneficial anti-biofouling effect was attributed to the influence of reactive oxygen species (ROS) generated in the presence of both NPs, in addition to the high reactivity of CuNPs for releasing toxic Cu^+2^ ions. The anti-adhesion capacity was observed in the membranes tested, presenting better results in the PA+0.25Cu-PSL membrane with significant differences compared to the PA+0.25Fe-PSL membrane. 

## Figures and Tables

**Table 1:** Surface properties of the membranes.

Membrane	Roughness (nm)	Contact Angle (°)
PA-PSL	5.7 ± 0.7	74.25 ± 6.47
PA+0.25Cu-PSL	39.2 ± 4.7	99.42 ± 6.13
PA+0.25Fe-PSL	70.3 ± 13.1	74.21 ± 1.55

**Table 2:** Desalination performance of membranes.

Membrane	Flux (l m^−2^ h^−1^ bar^−1^)	Reject (%)
PA-PSL	0.61 ± 0.27 ^a^	94.86 ± 0.68 ^a^
PA+0.25Cu-PSL	0.42 ± 0.16 ^a^	92.96 ± 2.79 ^a^
PA+0.25Fe-PSL	0.15 ± 0.03 ^b^	74.36 ± 4.57 ^b^

The mean with a different superindex has a difference at *p* < 0.01.

## References

[1] Gonzalez-Enriquez R., Armendariz-Ontiveros M.M. (2016). Evolución de la desalinización del agua: Tecnología y costos, Movimiento Ciudadano por el Agua. Primavera del.

[2] IDA (2017). Desalination Yearbook 2017–2018.

[3] Choi W., Jeon S., Kwon S.J., Park H., Park Y.-I., Nam S.-E., Lee P.S., Lee J.S., Choi J., Hong S. (2017). Thin film composite reverse osmosis membranes prepared via layered interfacial polymerization. J. Membr. Sci..

[4] Fimbres-Weihs G.A., Álvarez-Sánchez J., Villegas-Álvarez E. (2017). Fouling Modeling and Optimization of Membrane Module Design for Brackish and Seawater Desalination in the Mexican Pacific Coast: Project Description and Progress. Membranes.

[5] Bucs S.S. (2017). Biofouling in Reverse and Forward Osmosis Membrane Systems. Ph.D. Thesis.

[6] García Y., Quintero N., Vicencio B., Rodríguez D., Ozturk E., Mosquera T., Corrales U., Volkmann U.G. (2016). Influence of TiO_2_ nanostructures on anti-adhesion and photoinduced bactericidal properties of thin film composite membranes. RSC Adv..

[7] Pérez-Sicairos S., Miranda-Ibarra S., Lin-Ho S., Álvarez-Sánchez J., Pérez-Reyes J., Corrales-López K., Morales-Cuevas J. (2016). Membranas de nanofiltración, preparadas viá polimerización en interfase, dopadas con nanopartículas de ZnO: Efecto en su desempeño [Nanofiltration membranes, prepared via interface polymerization, doped with ZnO nanoparticles: Effect on their performance]. Rev. Mex. Ing. Quim..

[8] Lee S.Y., Kim H.J., Patel R., Im S.J., Kim J.H., Min B.R. (2007). Silver nanoparticles immobilized on thin film composite polyamide membrane: Characterization, nanofiltration, antifouling properties. Polym. Adv. Technol..

[9] Tamayo L., Azócar M., Kogan M., Riveros A., Páez M. (2016). Copper-polymer nanocomposites: An excellent and cost-effective biocide for use on antibacterial surfaces. Mater. Sci. Eng. C.

[10] Mulfinger L., Solomon S., Bahadory M., Jeyarajasingam A., Rutkowsky S., Boritz C. (2007). Synthesis and study of silver nanoparticles. J. Chem. Educ..

[11] Stefaniuk M., Oleszczuk P., Ok Y. (2016). Review on nano zerovalent iron (nZVI): From synthesis to environmental applications. Chem. Eng. J..

[12] Armendáriz-Ontiveros M.M., García A.G., Villalobos S.d., Weihs G.A.F. (2019). Biofouling performance of RO membranes coated with Iron NPs on graphene oxide. Desalination.

[13] Zhou L., Zhuang W., Wang X., Yu K., Yang S., Xia S. (2017). Potential effects of loading nano zero valent iron discharged on membrane fouling in an anoxic/oxic membrane bioreactor. Water Res..

[14] Palza H., Nuñez M., Bastías R., Delgado K. (2018). In situ antimicrobial behavior of materials having copper-based additives in a hospital environment. Int. J. Antimicrob. Agents.

[15] Cioffi N., Torsi L., Ditaranto N., Tantillo G., Ghibelli L., Sabbatini L., Bleve-Zacheo T., D’Alessio M., Zambonin P., Traversa E. (2005). Copper nanoparticle/polymer composites with antifungal and bacteriostatic properties. Chem. Mater..

[16] Aruoja V., Dubourguier H., Kasemets K., Kahru A. (2009). Toxicity of nanoparticles of CuO, ZnO and TiO_2_ to microalgae Pseudokirchneriella subcapitata. Sci. Total Environ..

[17] Meghana S., Kabra P., Chakraborty S., Padmavathy N. (2015). Understanding the pathway of antibacterial activity of copper oxide nanoparticles. RSC Adv..

[18] Kaweeteerawat C., Chang C., Roy K., Liu R., Li R., Toso D., Fischer H., Ivask A., Ji Z., Zink J. (2015). Cu nanoparticles have different impacts in Escherichia coli and Lactobacillus brevis than their microsized and ionic analogues. ACS Nano.

[19] Sarkar A., Das J., Manna P., Sil P. (2011). Nano-copper induces oxidative stress and apoptosis in kidney via both extrinsic and intrinsic pathways. Toxicology.

[20] García A., Rodríguez B., Oztürk D., Rosales M., Diaz D., Mautner A. (2017). Incorporation of CuO nanoparticles into thin-film composite reverse osmosis membranes (TFC-RO) for antibiofouling properties. Polym. Bull..

[21] Ben-Sasson M., Zodrow K.R., Genggeng Q., Kang Y., Giannelis E.P., Elimelech M. (2013). Surface functionalization of thin-film composite membranes with copper nanoparticles for antimicrobial surface properties. Environ. Sci. Technol..

[22] Ben-Sasson M., Lu X., Nejati S., Jaramillo H., Elimelech M. (2016). In situ surface functionalization of reverse osmosis membranes with biocidal copper nanoparticles. Desalination.

[23] Sarango L., Paseta L., Navarro M., Zornoza B., Coronas J. (2018). Controlled deposition of MOFs by dip-coating in thin film nanocomposite membranes for organic solvent nanofiltration. J. Ind. Eng. Chem..

[24] Rodríguez B., Oztürk D., Rosales M., Flores M., García A. (2018). Antibiofouling thin-film composite membranes (TFC) by in situ formation of Cu-(m-phenylenediamine) oligomer complex. J. Mater. Sci..

[25] Homayoonfal M., Mehrnia M.R., Shariaty-Niassar M., Akbari A., Ismail A.F., Matsuura T. (2014). A comparison between blending and surface deposition methods for the preparation of iron oxide/polysulfone nanocomposite membranes. Desalination.

[26] Liu Q., Yasunami T., Kuruda K., Okido M. (2012). Preparation of Cu nanoparticles with ascorbic acid by aqueous solution reduction method. Trans. Nonferrous Met. Soc..

[27] Baltazar S.E., García A., Romero A.H., Rubio M.A., Arancibia-Miranda N., Altbir D. (2014). Surface rearrangement of nanoscale zerovalent iron: The role of pH and its implications in the kinetics of arsenate sorption. Environ. Technol..

[28] Arancibia-Miranda N., Baltazar S.E., García A., Romero A.H., Rubio M.A., Altbir D. (2014). Lead removal by nano-scale zero valent iron: Surface analysis and pH effect. Mater. Res. Bull..

[29] Arancibia-Miranda N., Baltazar S.E., García A., Muñoz-Lira D., Sepúlveda P., Rubio M.A., Altbir D. (2016). Nanoscale zero valent supported by Zeolite and Montmorillonite: Template effect of the removal of lead ion from an aqueous solution. J. Hazard. Mater..

[30] Prabhu Y.T., Rao K.V., Sai V.S., Pavani T. (2017). A facile biosynthesis of copper nanoparticles: A micro-structural and antibacterial activity investigation. J. Saudi Chem. Soc..

[31] Khatami M., Heli H., Jahani P.M., Azizi H., Nobre M.A.L. (2017). Copper/copper oxide nanoparticles synthesis using Stachys lavandulifolia and its antibacterial activity. IET Nanobiotechnol..

[32] Marković D., Korica M., Kostić M., Radovanović Ž., Šaponjić Z., Mitrić M., Radetić M. (2018). In situ synthesis of Cu/Cu2O nanoparticles on the TEMPO oxidized cotton fabrics. Cellulose.

[33] Khan A., Rashid R., Younas R., Chong A. (2016). chemical reduction approach to the synthesis of copper nanoparticles. Int. Nano Lett..

[34] Mdlovu N.V., Chiang C.-L., Lin K.-S., Jeng R.-C. (2018). Recycling copper nanoparticles from printed circuit board waste etchants via a microemulsion process. J. Clean. Prod..

[35] Wang L., Hu C., Shao L. (2017). The antimicrobial activity of nanoparticles: Present situation and prospects for the future. Int. J. Nanomed..

[36] Janko C., Zaloga J., Pöttler M., Dür S., Eberbeck D., Tietze R., Lyer S., Alexiou C. (2017). Strategies to optimize the biocompatibility of iron oxide nanoparticles-“SPIONs safe by desing”. J. Magn. Magn. Mater..

[37] Kang G.-d., Cao Y.-m. (2012). Development of antifouling reverse osmosis membranes for water treatment: A review. Water Res..

[38] Kumar R., Ismail A. (2015). Fouling control on microfiltration/ultrafiltration membranes: Effects of morphology, hydrophilicity, and charge. J. Appl. Polym. Sci..

[39] Hurwitz G., Guillen G.R., Hoek E.M. (2010). Probing polyamide membrane surface charge, zeta potential, wettability, and hydrophilicity with contact angle measurements. J. Membr. Sci..

[40] Karlsson H., Cronholm P., Gustafsson J., Möller L. (2018). Copper Oxide Nanoparticles Are Highly Toxic: A Comparison between Metal Oxide Nanoparticles and Carbon Nanotubes. Chem. Res. Toxicol..

[41] Palza H. (2015). Antimicrobial polymers with metal nanoparticles. Int. J. Mol. Sci..

[42] Bataineh H., Pestovsky O., Bakac A. (2012). pH-induced mechanistic changeover from hydroxyl radicals to iron (IV) in the Fenton reaction. Chem. Sci..

[43] Chae H., Lee J., Lee C., Kim I., Park P. (2015). Graphene oxide-embedded thin-film composite reverse osmosis membrane with high flux, anti-biofouling, and chlorine resistance. J. Membr. Sci..

